# Using Wearable Sensors and Machine Learning to Automatically Detect Freezing of Gait during a FOG-Provoking Test

**DOI:** 10.3390/s20164474

**Published:** 2020-08-10

**Authors:** Tal Reches, Moria Dagan, Talia Herman, Eran Gazit, Natalia A. Gouskova, Nir Giladi, Brad Manor, Jeffrey M. Hausdorff

**Affiliations:** 1 Center for the Study of Movement, Cognition and Mobility, Neurological Institute, Tel Aviv Sourasky Medical Center, Tel Aviv-Yafo 6492416, Israel; talre@tlvmc.gov.il (T.R.); moriadagan@gmail.com (M.D.); talih@tlvmc.gov.il (T.H.); erang@tlvmc.gov.il (E.G.); nirg@tlvmc.gov.il (N.G.); 2 Sagol School of Neuroscience, Tel Aviv University, Tel Aviv 6997801, Israel; 3 Harvard Medical School, Boston, MA 02115, USA; NataliaGouskova@hsl.harvard.edu (N.A.G.); BradManor@hsl.harvard.edu (B.M.); 4 Hinda and Arthur Marcus Institute for Aging Research, Hebrew SeniorLife, Roslindale, MA 02131, USA; 5 Division of Gerontology, Beth Israel Deaconess Medical Center, Boston, MA 02215, USA; 6 Department of Neurology, Sackler Faculty of Medicine, Tel Aviv University, Tel Aviv 6997801, Israel; 7 Department of Physical Therapy, Sackler Faculty of Medicine, Tel Aviv University, Tel Aviv 6997801, Israel; 8 Rush Alzheimer’s Disease Center and Department of Orthopedic Surgery, Rush University Medical Center, Chicago, IL 60612, USA

**Keywords:** Parkinson’s disease, wearables, machine learning, freezing of gait, accelerometer, gyroscope

## Abstract

Freezing of gait (FOG) is a debilitating motor phenomenon that is common among individuals with advanced Parkinson’s disease. Objective and sensitive measures are needed to better quantify FOG. The present work addresses this need by leveraging wearable devices and machine-learning methods to develop and evaluate automated detection of FOG and quantification of its severity. Seventy-one subjects with FOG completed a FOG-provoking test while wearing three wearable sensors (lower back and each ankle). Subjects were videotaped before (OFF state) and after (ON state) they took their antiparkinsonian medications. Annotations of the videos provided the “ground-truth” for FOG detection. A leave-one-patient-out validation process with a training set of 57 subjects resulted in 84.1% sensitivity, 83.4% specificity, and 85.0% accuracy for FOG detection. Similar results were seen in an independent test set (data from 14 other subjects). Two derived outcomes, percent time frozen and number of FOG episodes, were associated with self-report of FOG. Bother derived-metrics were higher in the OFF state than in the ON state and in the most challenging level of the FOG-provoking test, compared to the least challenging level. These results suggest that this automated machine-learning approach can objectively assess FOG and that its outcomes are responsive to therapeutic interventions.

## 1. Introduction

Freezing of gait (FOG) is one of the most disabling and enigmatic phenomena that impacts people with Parkinson’s disease (PD) [1,2]. PD subjects with FOG suffer from restrictions in the movement of their feet, especially in stressful situations. FOG increases the risk of falling and impairs functional independence [3]. It can lead to the total arrest of the movement of the feet (i.e., akinesia); however, at other times, there is a kinetic manifestation in which the legs shake at a relatively high frequency, without the ability to move forward or complete a turn [2,4]. FOG tends to increase in frequency and severity with prolonged disease duration and progression of the disease [4]. While dopaminergic medications may reduce the incidence and severity of FOG, this symptom is still common in the ON (i.e., after medication intake) state, and it often does not respond favorably to treatment [4,5]. 

Because of its episodic nature and heterogeneous appearance, and because FOG often changes and may even vanish during an exam in the office or the laboratory [6,7], self-report has traditionally been the most widely used method for evaluating FOG and its severity [6,7,8]. The reliability and sensitivity to change are, however, not high for the existing self-report measures [7,9]. 

Another approach to assessing FOG is to use FOG-provoking tests. For example, Ziegler et al. [10] introduced a performance-based clinical test that includes several tasks known to trigger FOG, such as gait initiation, turning 360° on the spot (clockwise and counterclockwise), and passing through a doorway. The severity of FOG is rated by an expert observer based on a scale that reflects the occurrence of an observed FOG during predefined parts of the test. Although this scoring method helps to detect and assess FOG severity, it is, to some degree, subjective. Scoring of the test also requires clinical expertise, potentially limiting its widespread applicability. Furthermore, the number of episodes and FOG duration are not evaluated, and the detection of very short FOG episodes is difficult to quantify [11]. Tools enabling the objective assessment of FOG, the tracking of its progression, and the evaluation of the efficacy of related interventions are needed [6,8,12]. 

The objective assessment of FOG, theoretically, can be achieved with wearable inertial sensors. Inertial sensors can be placed at various body locations to obtain a complete picture of the patient’s movement [13,14]. Analyzing FOG episodes with inertial sensors enables objective measurement of FOG duration, its dynamics, the number of episodes, and the context in which they occurred [8,12,14,15]. This provides important additional information concerning FOG and, potentially, a better way of quantifying this problem.

Early works characterized FOG with metrics based in the time and frequency domain, either individually or combined, using conventional statistics [12,16,17,18,19,20]. More recently, several forms of supervised learning approaches were used to automatically detect FOG. These include support vector machines (SVM) [21,22,23,24,25], boosting ensembles [26,27], and trees [25,27]. Machine-learning (ML) classifiers make it possible to combine multiple features from different axes and sensors and to provide information in a predefined window length.

Although some FOG-detection algorithms based on ML and other techniques have already been investigated, work to date has largely been evaluated on relatively small datasets (e.g., the number of study participants ranged from 5 to 15 [22,23,26], less than 50 FOG episodes [26], or less than 100 min of FOG [21]) and independent test sets have not been used [22]. This limits generalizability. Another limitation of the extant work is that the clinometric properties of the output of the automated detection (e.g., response to intervention and association with other tests of FOG) often have not been examined.

Applying machine learning to wearable sensor data offers the possibility of automated detection of FOG that is not tuned to the individual and can be scored without clinical expertise. It has the potential to augment the existing evaluation of FOG in clinics and laboratory settings, similar to the way that the automatic sensor-based assessment of the Timed Up and Go (iTUG) has enhanced that test’s utility [28,29]. 

Leveraging a relatively large, manually annotated database of FOG, the aims of the present work were as follows: (1) to adapt an ML algorithm for the automated detection of FOG and to apply it, for the first time, to a previously validated FOG-provoking test; (2) to assess the ability of this ML algorithm to detect FOG episodes and to compare its detection performance to the freezing index and a previously published algorithm; (3) to evaluate the responsiveness of derived FOG outcomes to conditions known to affect FOG severity (i.e., how the derived outcomes change in ON versus OFF medications and how they change in simple versus more complex levels of the FOG-provoking test); and (4) to further explore the validity of derived FOG outcomes by evaluating their associations to other available methods for assessing FOG (e.g., the New FOG Questionnaire (NFOGQ) and Movement Disorder Society - Unified Parkinson’s Disease Rating Scale (MDS-UPDRS) part III).

## 2. Materials and Methods

### 2.1. Participants

The current work is based on a dataset that was originally collected as part of another study. That study aimed to examine the impact of transcranial direct current brain stimulation (tDCS) on FOG in individuals with PD (manuscript in preparation). Seventy-one PD subjects with FOG were selected from the dataset. They were recruited from the Center for the Study of Movement, Cognition, and Mobility (CMCM) at the Neurological Institute in Tel Aviv Sourasky Medical Center, Israel, and the Hinda and Arthur Marcus Institute for Aging Research, Hebrew SeniorLife, Harvard Medical School, Boston, MA. All study participants met the UK Brain Bank criteria for idiopathic PD. Additional inclusion criteria included the following: (1) a minimum of 21 points on the Mini Mental State Examination [30], (2) a minimum score of 6 on the NFOGQ [31], (3) age 40–95 years old, and (4) a stable medication regimen, i.e., no change in medications for the month prior to study participation. Exclusion criteria were as follows: (1) inability to walk independently, (2) self-report of neurological or psychiatric disorder other than PD, (3) history of seizures or head trauma, and (4) implanted metals in the head area. All subjects provided informed written consent, as approved by local human studies committees.

### 2.2. FOG-Provoking Test and Wearable Sensors

**Figure 1:**
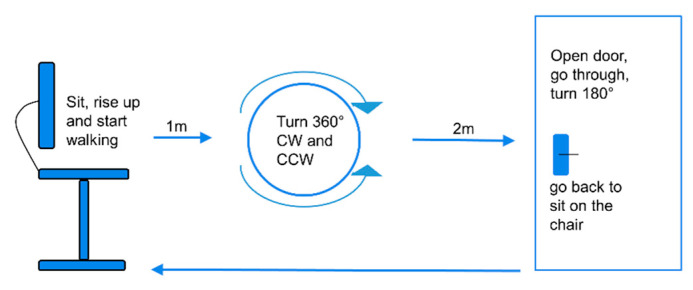
A scheme of the freezing of gait (FOG)-provoking test the participants conducted in the lab [10]. The test was repeated three times, with three different levels of difficulty. From a seated position, the subject walks, turns in a circle clockwise and counter clockwise, as indicated, enters a doorway, turns, and then returns to the seated position. CW, clockwise; CCW, counterclockwise.

The study participants performed a FOG-provoking test in the lab (see Figure 1 for an overview of the test) [10]. Each patient performed the test at three levels of difficulty: (1) as a single task, (2) as a dual-motor task, i.e., performing the trial while carrying a tray with a bottle on it, and (3) as a motor–cognitive task. In this most-challenging level, the participants were asked to perform the trial as described in the second level of difficulty, while also performing serial seven subtractions. The subjects were assessed while wearing Opal inertial sensors (APDM, Inc., Portland, OR, USA) on the lower back and at the side of each leg, above the ankle. Each sensor included a tri-axial accelerometer, gyroscope, and magnetometer recording at 128 Hz. The signals from the lower-back sensor provide insight into the left and right feet, as well as the trunk and upper body, and the signals from the ankle sensors provide more detailed information regarding movement of the legs. The subjects were tested before the administration of tDCS or sham, and again in three different visits after the administration of tDCS or sham (for more study details see ClinicalTrials.gov protocol NCT02656316). In each visit, the test was performed in the OFF state (at least 12 h with anti-parkinsonian medication withdrawal) and/or in the ON state and included the three levels mentioned above.

As a “gold standard” reference in order to be able to validate the output of the ML algorithm, each test was videotaped and annotated offline for any FOG episodes that occurred. An in-house FOG tagging (annotation) program was developed for this purpose as a MATLAB GUI; it enabled synchronization between the videos and the signals and facilitated the annotation process. Predefined guidelines were used for manual detection of FOG [5,20,32]. Briefly, FOG was defined as an intention to walk without movement of the feet, or as heel lifting while toes stay on the ground, or an irregular turning rhythm while the pivot foot stays on the ground. To reduce inter-rater variability, all videos of the same individual were annotated by the same assessor. Videos that were noted with no FOG episodes or that were difficult to annotate due to very short episodes or akinesia, for example, were reviewed by two additional assessors. Preprocessing of the labeled dataset included merging separately identified FOG episodes that occurred <1 second apart, as well as assigning identified FOG episodes lasting shorter than one second in duration to a non-FOG segment.

### 2.3. Machine-Learning Algorithms

An automatic FOG-detection model was created by using ML methods. A support vector machine (SVM) with radial basis function (RBF) kernel was chosen as the classification method. This classifier has shown good results among previous publications of FOG detection [21,22,24,25,33]. Fourteen subjects (20%) were selected randomly as the test set, and the other 57 subjects (80%) were selected for the training set. All the date was first filtered with a second-order Butterworth low-pass filter with a cutoff frequency of 15 Hz, to reduce high frequency noise. The training set was divided into three-second windows with 50% overlap for non-FOG windows and 80% overlap for FOG windows, thus creating more FOG examples and improving the model’s performance. For classification, only a window that contained at least 1.5 s of FOG episodes was considered as a FOG window; otherwise, it was considered to be a non-FOG window. For testing the model, the test set was partitioned into three-second windows with 50% overlap. The windows were overlapped to achieve maximum information and fine-tuning of the temporal resolution.

**Figure 2:**
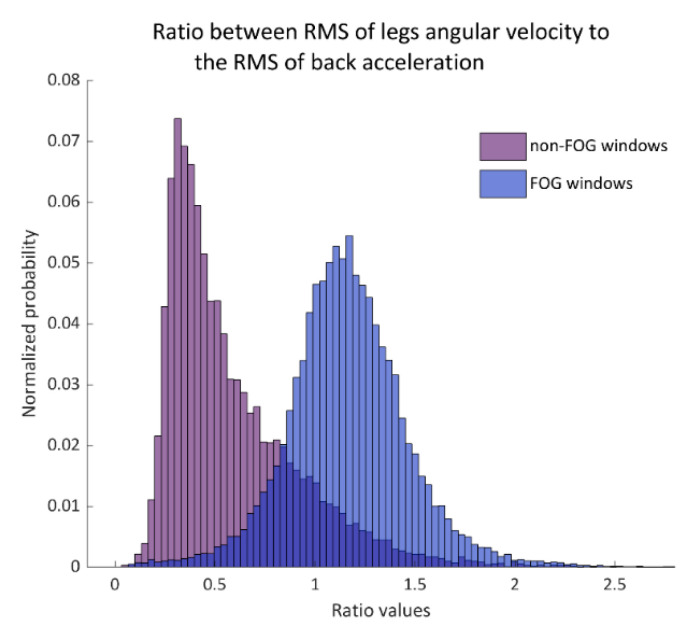
The histogram of the ratio between legs gyroscope root-mean-square (RMS) to the back-acceleration RMS in non-FOG windows (left histogram) and FOG windows (right histogram). This ratio was used as a primary input feature to the support vector machines (SVM) model. Probability is shown in normalized values.

A set of 86 features was computed for each window and from several sensors (see Table A1 in the Appendix A). The feature set was based on previous publications [21,22,23] and was adapted and modified to the current dataset (e.g., the axes and frequency bands that were used). Two features that showed the relationship between the movements of the back to that of the legs were added. Figure 2 demonstrates one of these features. Feature selection was performed to identify the most discriminative features for distinguishing FOG windows from non-FOG windows. This was done through a leave-one-patient-out method in which each of the iterations was repeated by keeping apart the data from a single patient. This way the model was tested with data that were not included in its training and a model with a final set of features could be obtained. 

Feature selection in each leave-one-patient-out iteration was done as follows; t-tests were used within each feature, and only the features that significantly separated between windows with and without FOG with corrections to multiple comparisons (Bonferroni) were retained. Next, the selected features were ranked by their relevance, using the minimum redundancy, maximum relevance (MRMR) algorithm [34]. Finally, an iterative feature-selection process was performed in which the misclassification error as a function of the number of the ranked features was analyzed. The feature set that gained the minimum misclassification error was chosen. 

Furthermore, the classification validation was performed with the leave-one-patient-out method. The data from a single patient that were kept out of the training batch were tested, and each window was compared to the reference annotations. The performance of each of the iterations in the detection of FOG was measured by sensitivity, specificity, and accuracy in a window level. When there were no FOG windows in the signal, sensitivity was considered to be 100%. A ROC (receiver operating characteristic) curve was calculated for each iteration. Afterward, the means across all the iterations of sensitivity, specificity, and accuracy, as well as the mean ROC curve, were calculated.

**Figure 3:**
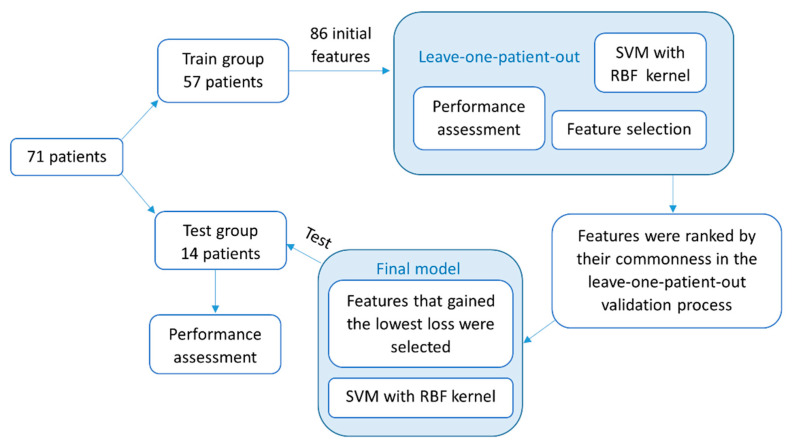
A flowchart of the methodology described in this paper for the detection of FOG episodes with a machine-learning classifier. First described are the feature-selection and -validation processes in a leave-one-patient-out method. Then, the construction and testing of the final model. SVM, support vector machine; RBF, radial basis function.

A final model was generated with the training set of 57 subjects and tested on the dataset of 14 other subjects who were not included in the training set. The previously described iterative feature-selection process was used in the final model. However, first, the features were ranked according to their commonness in the previously described leave-one-patient-out process. Then, the final set of features was selected according to the minimum classification error that was achieved among the training group and was used as an input to the final classifier. Automatic FOG detection was made by using the final model, and accuracies were achieved. Finally, each patient’s dataset was evaluated for the number of FOG episodes and the total duration of FOG. An episode’s duration was quantified by its ratio to the test’s duration and referred to as “percent time frozen” [12]. See Figure 3 for a flowchart of the classification process.

Two other algorithms were additionally evaluated for comparison: the freezing index [16], as a single measure, and the feature set suggested by Samà et al. [23]. The windowing process for both was the same as for the new suggested algorithm. The freezing index, originally developed by Moore et al. [16], is a threshold-based method to identify FOG. It is based on the idea that the power spectrum of FOG differs from that of normal gait [35]. The freezing index was calculated for each window as the ratio between the integrated power spectral density in 3–8 Hz (freeze band) to that in 0.5–3 Hz (walking band). The anterior–posterior acceleration *axis* was used for this purpose [17], and a threshold of 2.5 (AU) was determined for the detection of FOG. The freezing index was calculated for each leg separately, and the maximum value between the legs was chosen. The results were examined as described earlier with sensitivity, specificity, and accuracy per window. The second feature set that was implemented (Samà et al. [23]) was used as an input for the SVM classifier (see Table 1 for details). This feature set was chosen as a starting point because it showed promising results in detection with ML methods. The feature set included cross-axis information as correlations and differences in the mean value of the axes.

### 2.4. Statistical Analysis

Statistical analyses were performed by using SPSS statistical package version 25 (SPSS Inc., Chicago, IL), and MATLAB, 2019 version 9.7.0 (R2019B) (Natick, MA: The MathWorks, Inc.). A 2-tailed *p*-value ≤ 0.05 was considered statistically significant. Sensitivity was calculated as the sum of the true positive (FOG) windows, as detected by the algorithm divided by the total amount of FOG windows, according to video annotations. Specificity was calculated as the sum of the true negative (non-FOG) windows, as detected by the algorithm divided by the total amount of non-FOG windows, according to video annotations. Accuracy was the ratio between the sum of the true positive detections and true negatives detections to the entire population. For the train-test process of the classifier, the entire dataset was used. To study the responsiveness (e.g., change in the outcome measures in response to a challenging condition) and associations of the output of the algorithm, we focused on the baseline data (before the administration of tDCS) in order not to be influenced by the tDCS treatment on the existence of FOG. A square-root transformation was used to achieve normal distribution for the average of percent time frozen, the number of FOG episodes, and the clinical test score across the three levels of the FOG-provoking test. Paired t-tests were used to investigate differences between the OFF- and ON-medication states. Friedman’s tests were used to investigate differences between the test levels within medication state condition, and Wilcoxon signed-rank tests were used to investigate the post hoc analysis. Wilcoxon singed-rank tests were also used to investigate differences between the performance of the new algorithm and two other methods. The performance of the three methods was measured in terms of sensitivity, specificity, and accuracy. Effect sizes for paired-sample t-tests (*d*) were calculated by dividing the mean difference by the standard deviation of the difference: *d* = 0.2 small effect, *d* = 0.5 medium effect, and *d* = 0.8 strong effect [36]. Effect sizes for Wilcoxon signed-rank tests (r) were calculated by dividing the test statistic by the square root of the number of observations: *r* = 0.1 small effect, *r =* 0.3 medium effect, and *r* = 0.5 and higher strong effect [36]. The associations between the algorithm-based measures and annotations-based measures, as well as other tests that reflect FOG severity, were calculated with Spearman’s correlations. 

## 3. Results

Table 2 summarizes the characteristics of the study participants. In general, the study participants had relatively advanced disease and relatively high scores on the NFOGQ, consistent with the inclusion criteria.

### 3.1. Detection Performance

For the classification process, 1041 recorded signals with a total duration of 17.6 h were recorded. Based on the video annotations, 1754 FOG episodes were identified before merging episodes that were close to each other (<1 s), and 1487 were identified after merging. The total FOG duration across all records was 6.75 h, with 0 to 11 episodes per test. The single-episode duration ranged from 0.18 s before eliminating short episodes (<1 s) to a maximum of 11.46 min. The training set (57 subjects) consisted of 12,629 windows of FOG (average of 550 windows per subject) and 20,172 non-FOG windows (average of 340 windows per subject) based on the manual video annotations. Each signal could have both FOG and non-FOG windows. 

**Figure 4:**
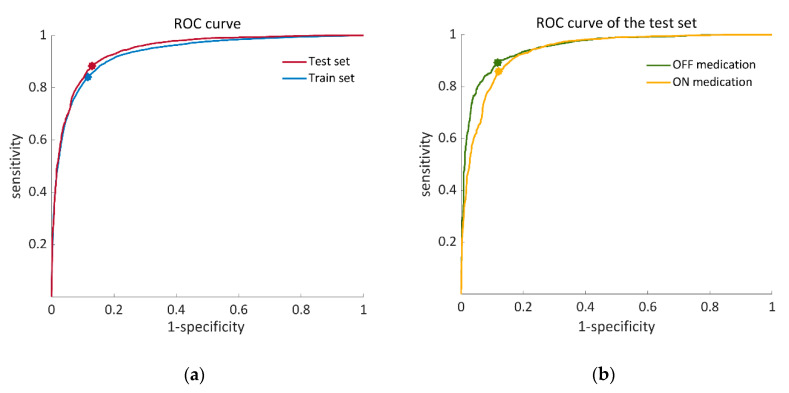
ROC (receiver operating characteristic) curves that illustrate the performance of the proposed SVM classifier. (**a**) Training set (blue) calculated from 57 iterations of a leave-one-patient-out validation process, AUC = 0.93, OOP: (0.12, 0.84). Test set (red), AUC = 0.94, OOP: (0.13, 0.88); (**b**) ROC curves of the OFF–ON states in the test set. OFF state (green), AUC = 0.95 OOP: (0.12, 0.89). ON state (yellow), AUC = 0.94: OOP: (0.12, 0.86). AUC, area under the curve; OOP, optimal operation point.

Following the feature-selection process, 18 features were used per training iteration on average. The average results were 84.1 ± 22.3% sensitivity, 83.4 ± 12.2% specificity, and 85.0 ± 10.0% accuracy. The average ROC curve across all the iterations had an AUC = 0.93 for the training set (see Figure 4a).

For the final classification model in which the dataset of 57 individuals was trained by the SVM classifier, 14 important features were chosen (Table 3) out of a list of 86 features (Table A1 in the Appendix A). The test set of 14 subjects showed 80.0 ± 19.2% sensitivity, 82.5 ± 11.2% specificity, and 86.6 ± 7.8% accuracy. The AUC of the ROC curve of the test set was 0.94 (see Figure 4a). Figure 4b illustrates ROC curves of the test set separately for the OFF- and ON-medication states. In addition to that, algorithm-derived percent time frozen during the test set was significantly correlated with the annotations-derived percent time frozen (r = 0.897 *p* < 0.01).

**Figure 5:**
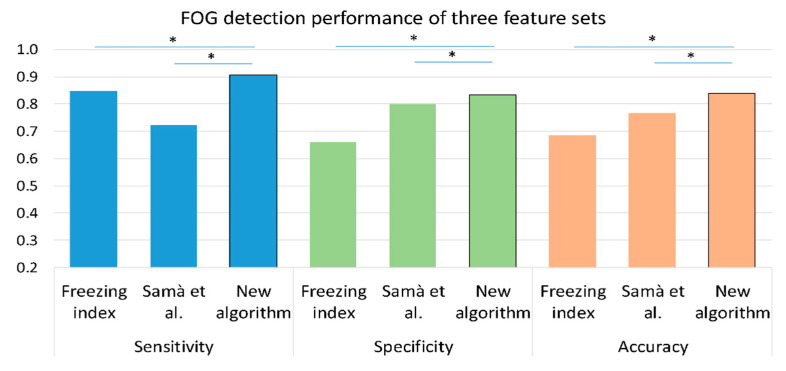
A comparison between the performance of three methods in the detection of FOG episodes on the training set (*n* = 57); freezing index as a single feature, Samà et al. [23] feature set with an SVM classifier and the proposed feature set with the same SVM classifier. The training set was chosen for this comparison since it consists of a larger dataset. Similar results were obtained with the test set. * *p* < 0.05, values are presented as median.

Figure 5 presents the sensitivity, specificity, and accuracy of the freezing index as a single feature and of an SVM with the feature set from Samà et al. [23], as well as the results of the proposed algorithm. As shown in the figure, the proposed algorithm had better accuracy, sensitivity, and specificity compared to the two previously used algorithms.

### 3.2. Responsiveness of the Assessment of FOG Based on the Outcomes of the Algorithm

**Figure 6:**
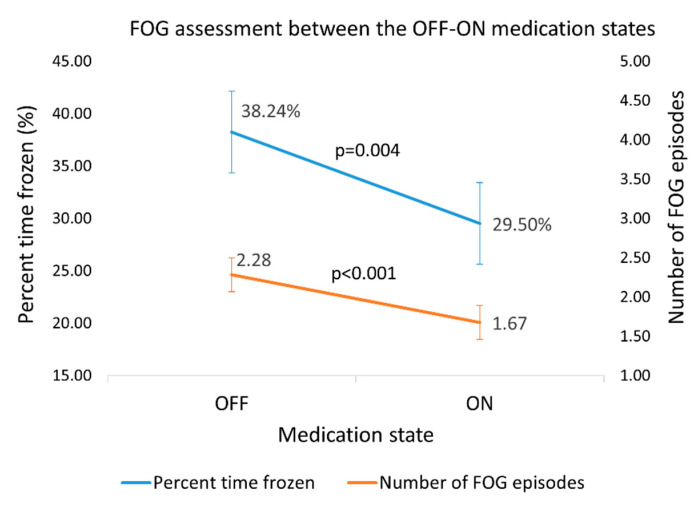
Percent time frozen and the number of FOG episodes, as detected by the new algorithm in the OFF–ON states. Error bars reflect ± 1 standard errors.

The dataset (71 subjects) was evaluated by the proposed algorithm for the quantity and extent of FOG episodes. As expected, paired t-tests showed that percent time frozen and the number of episodes averaged across the three levels of the test were higher in the OFF state than in the ON state (*n* = 43 subjects, %FOG: *p* = 0.004, effect size = 0.5; number of episodes: *p* < 0.001, effect size = 0.6) (Figure 6). Similarly, the clinical score on the FOG-provoking test increased (*p* = 0.002) from 4.0 ± 2.2 in the ON state to 5.1 ± 2.3 in the OFF state, with an effect size of 0.5.

Furthermore, Friedman’s tests revealed significant differences in the FOG measures between the levels of difficulty both in OFF and ON (OFF; *n* = 41 subjects, %FOG: *p* = 0.011, number of episodes: *p* < 0.001. ON; *n* = 62 subjects, %FOG, and number of episodes: *p* < 0.001). Post hoc analysis of the test levels in OFF–ON states showed that the highest percent time frozen was in the most challenging test level (Table 4).

### 3.3. Associations of Algorithm Measures of FOG with Related Measures of FOG 

Correlations between the algorithm’s estimate of FOG and self-report of NFOGQ score, disease duration, TUG time, and MDS-UPDRS part III in OFF and ON are presented in Table 5. Mild-to-moderate correlations were observed, with some of the associations being dependent on the medication state.

## 4. Discussion and Conclusions

Here we describe a machine-learning method for the automatic detection of FOG episodes during a FOG-provoking test [10] using data from three wearable sensors. The FOG-provoking test included conditions that aimed to trigger FOG presentation, and thus it involves both walking and turning, as well as cognitive, motor, and emotional challenges. As was recently described in Reference [9], objective ways of evaluating FOG are needed. To the best of our knowledge, this is the first study that aimed to automatically detect FOG episodes during a previously validated FOG-provoking test. Several findings support the utility and validity of the present approach: (1) the relatively high sensitivity and specificity results of the training and, maybe, more importantly, the test set; (2) correlations between the ML-based outcomes and the manual video review of FOG; (3) the responsiveness of derived measurements from the automated detection to anti-parkinsonian medications and the more challenging FOG-provoking condition; and (4) the correlations between algorithm-derived measures of FOG and the NFOGQ. 

Recent studies underline the importance of using relatively large datasets for studying FOG detection and specifically for ML approaches [6,9,13]. For example, a 2019 review screened 68 papers that aimed to detect FOG [13], and the largest studied population was only 32 FOG-PD subjects. In the present study, records from 71 subjects with a total of 6.75 hours of FOG episodes were included. This reflects a relatively large number of annotated FOG episodes with prolonged total duration, consistent with the recent recommendations. 

As suggested before [21,22,23], we found that patient-independent SVM classifiers can be used to detect FOG. The statistical measures of the performance of the classifier and the high correlations between the algorithm-derived results and the actual percent time frozen (r = 0.897, *p* < 0.01) indicates the good performance of the algorithm. The AUC reinforces this conclusion, specifically the similarity between the ROC curves of the OFF–ON medication states (Figure 4b). The minor differences between the OFF–ON curves likely reflect false negatives, due to a decrease in the occurrence of FOG in the ON state. Furthermore, there was some variation in the detection accuracy among the tested subjects, as can be seen by the standard deviations of the algorithm performance matrices. This might be due to the variations in the manifestations of FOG during the test across individuals. FOG was previously described as a mysterious symptom that is challenging to observe, which is manifested differently among individuals with PD, and that even changes in different situations for the patient him/herself [2]. Episode duration ranged between 1.0 to 687.6 s, and very short episodes were challenging for the clinicians to annotate and for automatic detection. Indeed, the present analyses did not consider FOG that was less than one-and-a-half-seconds long (due to a window length of 3 s)—a limitation. Akinesia, impaired gait, akinetic episodes, and deceleration (preceding opening the door, for example) likely also challenged the automated detection. Promisingly, the sensitivity and specificity of the detection of FOG episodes among the test set were very close to the performance of the training process. This suggests that the classifier was not over-trained or tuned to the specifics of the study participants and that it is generalizable.

The feature-selection process reduced the feature count from the original 86 to 14 features in total (Table 3), without compromising detection performance. This can make the classification process more accessible for further use. It is also important to note that we introduced new features: the ratio between the RMS from the leg sensors to the RMS from the back sensor (angular velocity from legs to acceleration from the back and angular velocity of both) (recall Figure 2). These features were based on the idea that, during kinetic freezing, the movement of the body forward decreases while the legs are still moving. This notion was supported by the high rank that these features received by the MRMR algorithm as part of the feature-selection process. It can be seen that most of the features that were included in the final model input were calculated from the leg sensors (Table 3). This finding may be because the manifestation of FOG decreases as the sensor moves away from the ground.

FOG duration and episode counts may have an added value in the evaluation of the FOG-provoking test that is usually scored only by the presence of episodes in predefined tasks. As expected, since FOG occurs more frequently in the OFF state and in more challenging tasks [2], the percent time frozen and the number of episodes were higher in the OFF than in ON state (Figure 6), and in the most challenging level of the test than the easiest level (Table 4). These findings support the validity of these derived outcome measures. Interestingly, the effect sizes of the clinical score and FOG measures were similar (recall Table 5). We can infer from this that the automated assessment performs as well as the clinical rating. Of course, evaluating percent time frozen and the number of episodes in a FOG-provoking test can be accomplished by manual review of video-recorded tests. However, that rating process is quite time-consuming, requires trained personnel, and is subject to bias. The present findings indicate that the automated process based on wearable sensors and machine learning can be used instead of the manual review of videos.

To directly compare the results of the detection performance with the literature, we tested two additional methods of FOG detection. Figure 5 shows that the results based on those methods were lower than those of the new algorithm. Although the freezing index was one of the main input features in our classifier, as a single feature, its performance was insufficient. Samà et al. [23] presented results that are higher than those achieved by their same features with our dataset. That might be a consequence of the location of the sensors and specific characteristics of the dataset. The FOG-provoking test was made of short-distance trials with turns and few walking bouts, compared to the datasets from both studies that contained longer recordings of free walking. Difficulties in reproducing the results of previously published methods of automatic FOG detection were already described [23,26,37]. More specifically, the importance of validation of ML methods on different cohorts was recently highlighted [6]. Many factors may influence the ability to generalize one method to different datasets. These include the characteristics of the participants, the placement of the sensors, the preprocessing of the data, and the evaluation methods of the results. Caution should be used when trying to compare and utilize the automatic FOG detection to different cohorts with different conditions. Nonetheless, our use of a training set and a completely different test set should mitigate this issue.

Accuracy, sensitivity, and specificity above 80% in this context can be considered good. Still, for some applications, it might be helpful to increase detection performance. This might be achieved by adding additional sensor types (e.g., EMG [38] and ECG [39]) to help differentiate some of the more challenging conditions (e.g., akinetic FOG vs. standing still), by focusing on the subtypes of episodes (e.g., during a turn and start hesitation) or by applying techniques like deep learning [37]. The present work can also be extended to develop an automated and instrumented scoring. A total score might be based on the percent time frozen and the number of episodes, and perhaps sub-scores might relate to the different FOG-provoking test parts, e.g., gait initiation, turns, and straight-line walking. This might enhance monitoring and provide directions for therapy. Furthermore, it might be interesting to combine the objective score with other sensor-derived measures (e.g., duration of subtasks), self-report (e.g., NFOGQ score), and other FOG characteristics that are already known [40]. Reducing the number of sensors to achieve simplicity, especially for future use for home and daily living detection, should also be considered. Trade-offs between simplicity and ease-of-use, on the one hand, and complexity and granularity of the tool, on the other hand, will need to be evaluated.

The present results suggest that the automated detection of FOG within a laboratory-based FOG-provoking test can be achieved by using wearables and a machine-learning algorithm. This tool promises to augment the assessment of this debilitating phenomenon and, hopefully, aid in the development of improved treatments by affording objective evaluation of their impact on FOG.

## Figures and Tables

**Table 1:** The feature set according to Samà et al. [23]. Features were calculated from the back sensor.

Feature	Axis/Frequency
**Time domain**	
Difference of the mean	AP, V-ML, AP-ML
SD	AP, V, ML
Correlation	V-AP, AP-ML, V-ML
Skewness	AP, V, ML
Skewness of the RMS	.
**Frequency domain** All features were calculated for the sum of the three axes vectors	
SD in different bands	0.04–0.68 Hz, 0.68–3 Hz, 3–8 Hz, 8–20 Hz, 0.1–8 Hz
Max harmonic and its frequency	.
Distance between the first and second max harmonics	.
Center of Mass	.
Skewness in different bands	0.04–0.68 Hz, 0.68–3 Hz, 3–8 Hz,
First three components of PCA	0.04–8 Hz

AP, anterior-posterior *axis*; V, vertical *axis*; ML, medio-lateral *axis*; RMS, root-mean-square; SD, standard deviation; PCA, principal component analysis.

**Table 2:** Subject characteristics.

**No. of subjects** * (N)	71
**Age** (years)	69.9 ± 7.8
**Gender** (M:F)	(57:14)
**Disease duration** (years)	9.2 ± 5.7
**Education** (%)	22% High school or equivalent 23.9% Bachelors 32% Masters or higher
**New FOG Questionnaire**	19.4 ± 4.3
**Mini Mental Status Exam**	28.0 ± 1.8
**MDS-UPDRS part III (motor) OFF**	43.1 ± 16.9
**MDS-UPDRS part III (motor) ON**	37.1 ± 14.5
**TUG time OFF** (s)	15.3 ± 10.2
**TUG time ON** (s)	13.6 ± 7.9
**FOG-provoking test total score OFF**	15.8 ± 7.0
**FOG-provoking test total score ON**	12.5 ± 6.6
**Gait speed** (cm/s) **OFF**	100.3 ± 22.8
**Gait speed** (cm/s) **ON**	104.6 ± 25.2

* Forty-four subjects were tested both in OFF and ON states. Twenty-four were tested only in the ON state, and three only in the OFF state. MDS-UPDRS, Movement Disorder Society Unified Parkinson’s Disease Rating Scale.

**Table 3:** A set of 14 features that were used to train the final model with the SVM classifier. These features yielded the minimum classification loss and are rated in descending order, according to their significance to the model.

	**Domain**	Feature Description	Sensor Location	Accelerometer/Gyroscope
1	Time	$\frac{\max\left(\mathrm{RMS}\;\left(x_{\mathrm{RL}},y_{\mathrm{RL}},z_{\mathrm{RL}}\right),\mathrm{RMS}\;\left(x_{\mathrm{LL}},y_{\mathrm{LL}},z_{\mathrm{LL}}\right)\right)\;}{\mathrm{RMS}\;\left(x_{\mathrm{back}},y_{\mathrm{back}},z_{\mathrm{back}}\right)}$	Legs and back	Legs: Gyroscope, Back: Acceleration, (x,y,z)∈{V,AP,ML}
2	Frequency	Freezing index (x)	Legs	Acceleration, $x\in {\mathrm{AP}}_{0.5–3\;\mathrm{Hz},\;3–8\;\mathrm{Hz}}$
3	Frequency	Peak frequency of x between 3–8 Hz	Legs; max between both legs	Acceleration, $x\in V_{3–8\;\mathrm{Hz}}$
4	Frequency	Entropy of x between 0.5–3 Hz	Back	Gyroscope, $x\in V_{0.5–3\;\mathrm{Hz}}$
5	Frequency	Peak frequency of x between 0.5–3 Hz	Legs; ${\max(x}_{\mathrm{RL}},x_{\mathrm{LL}})$	Gyroscope, $x\in V_{0.5–3\;\mathrm{Hz}}$
6	Time	Range(cumulative sum (x))	Back	Gyroscope, x∈ML
7	Frequency	Entropy of x in specific bands 0.5–3 Hz	Back	Acceleration, $x\in {\mathrm{AP}}_{0.5–3\;\mathrm{Hz}}$
8	Time	Range(cumulative sum (x))	Legs; min between both legs	Acceleration, x∈V
9	Time	Mean (x)	Legs; $\frac{\min\left(\left|{\bar{x}}_{\mathrm{RL}}|,|{\bar{x}}_{\mathrm{LL}}\right|\right)}{\max\left(\left|{\bar{x}}_{\mathrm{RL}}|,|{\bar{x}}_{\mathrm{LL}}\right|\right)}$	Gyroscope, x∈ML
10	Frequency	Skewness of x between 0.5–3 Hz	Back	Gyroscope, $x\in {\mathrm{ML}}_{0.5–3\;\mathrm{Hz}}$
11	Time	Correlations between the right and left leg	Legs; $\mathrm{corr}\left(x_{\mathrm{RL}}{,x}_{\mathrm{LL}}\right)$	Gyroscope, x∈ML
12	Time	$\frac{\max\left(\mathrm{RMS}\;\left(x_{\mathrm{RL}}{,y}_{\mathrm{RL}}{,z}_{\mathrm{RL}}\right),\mathrm{RMS}\;\left(x_{\mathrm{LL}}{,y}_{\mathrm{LL}}{,z}_{\mathrm{LL}}\right)\right)\;}{\mathrm{RMS}\;\left(x_{\mathrm{back}}{,y}_{\mathrm{back}}{,z}_{\mathrm{back}}\right)}$	Legs and back	Gyroscope, (x,y,z)∈{V,AP,ML}
13	Time	Range(cumulative sum (x))	Legs; ${\min(x}_{\mathrm{RL}}{,x}_{\mathrm{LL}})$	Gyroscope, x∈ML
14	Time	RMS(x,y,z)	Legs; ${\max(x}_{\mathrm{RL}}{,x}_{\mathrm{LL}})$	Gyroscope, (x,y,z)∈{V,AP,ML}

RL, right leg; LL, left leg; RMS, root-mean-square; V, vertical *axis*; AP, anterior-posterior *axis*; ML, medio-lateral *axis*; corr, correlation.

**Table 4:** FOG outcomes determined by using the automated algorithm differ in between the easiest and most challenging levels of the test within OFF–ON states.

	No. of Subjects	Easiest Level	Most Challenging Level	Effect Size	*p*-Value
OFF medication	41				
Percent time frozen (%)		35.7 (7.2–51.1)	36.5 (17.4–69.8)	0.4	0.017
Total time frozen (s)		15.0 (3.0–24.8)	24.0 (9.0–69.8)	0.6	<0.001
Number of FOG episodes		1.0 (1.0–3.0)	3.0 (1.5–4.0)	0.7	<0.001
ON medication	62				
Percent time frozen (%)		21.0 (0–43.4)	37.8 (11.2–50.4)	0.6	<0.001
Total time frozen (s)		9.0 (0.0–20.3)	18.0 (4.5–38.1)	0.7	<0.001
Number of FOG episodes		1.0 (0.0–2.0)	2.0 (1.0–4.0)	0.7	<0.001

Values are presented as median (inter-quartile range). Since some of the subjects were not able to perform the testing in the OFF state, the number of subjects was higher in the ON state.

**Table 5:** Spearman correlations of percent time frozen, as detected by the algorithm and NFOGQ score, TUG time, and MDS-UPDRS part III score in OFF–ON medication states.

	NFOGQ Total	TUG Time	MDS-UPDRS Part III	Disease Duration
OFF medication				
Percent time frozen (%)	0.489 **	0.263	0.074	−0.253
Total time frozen (s)	0.485 **	0.392 **	0.116	−0.176
Number of episodes	0.391 **	0.420 **	0.210	0.029
ON medication				
Percent time frozen (%)	0.375 **	0.379 **	0.496 **	−0.042
Total time frozen (s)	0.405 **	0.471 **	0.565 **	−0.007
Number of episodes	0.416 **	0.583 **	0.565 **	0.071

Significant correlations are bolded: ** *p* < 0.01. TUG, Timed Up and Go; NFOGQ, New FOG Questionnaire. MDS-UPDRS, Movement Disorders Society Unified Parkinson’s Disease Rating Scale.

## Data Deposition Information

The data will be made available from the authors upon reasonable request.

## Appendix A

**Table A1:** The full feature set (84 features) that was used in the machine-learning training.

No. of Features	Feature Description	Back Sensor	Leg Sensors
**Time domain**			
6	Mean(x)	Acceleration, x∈{V,AP}	$\frac{\min\left(\left|{\bar{x}}_{\mathrm{RL}}|,|{\bar{x}}_{\mathrm{LL}}\right|\right)}{\max\left(\left|{\bar{x}}_{\mathrm{RL}}|,|{\bar{x}}_{\mathrm{LL}}\right|\right)}$ $\left|{\bar{x}}_{\mathrm{RL}}-{\bar{x}}_{\mathrm{LL}}\right|$ Acceleration, x∈V Gyroscope, x∈ML
6	SD(x)	Acceleration, x∈{V,AP}	$\max\left(\left|\mathrm{SD}\left(x_{\mathrm{RL}}\right)\right|,\left|\mathrm{SD}\left(x_{\mathrm{LL}}\right)\right|\right)$ $\left|{\bar{x}}_{\mathrm{RL}}-{\bar{x}}_{\mathrm{LL}}\right|$ Acceleration, x∈V Gyroscope, x∈ML
6	Correlations(x, y)	Acceleration, (x, y)∈{V-ML, V-AP, AP-ML}	$\max(\mathrm{corr}\left(x_{\mathrm{RL}}{,y}_{\mathrm{RL}}\right),\mathrm{corr}\left(x_{\mathrm{LL}}{,y}_{\mathrm{LL}}\right))$ Gyroscope, (x, y)∈{V-ML, V-AP, AP-ML}
1	Correlations between the right and left leg	.	$\mathrm{corr}\left(x_{\mathrm{RL}}{,x}_{\mathrm{LL}}\right)$ Gyroscope, x∈ML
7	Range(cumulative sum (x))	Acceleration, x∈{V,AP} Gyroscope: x∈ML	$\min\left(\begin{array}{c} \mathrm{range}\left(\mathrm{cumulative}\;\mathrm{sum}\left(x_{\mathrm{RL}}\right)\right),\;\\ \mathrm{range}\left(\mathrm{cumulative}\;\mathrm{sum}\left(x_{\mathrm{LL}}\right)\right) \end{array}\right)$ $\max\left(\begin{array}{c} \mathrm{range}\left(\mathrm{cumulative}\;\mathrm{sum}\left(x_{\mathrm{RL}}\right)\right),\;\\ \mathrm{range}\left(\mathrm{cumulative}\;\mathrm{sum}\left(x_{\mathrm{LL}}\right)\right) \end{array}\right)$ Acceleration, x∈V Gyroscope, x∈ML
2	RMS (x)	Acceleration, x∈{V,AP}	.
2	RMS (x,y,z); total RMS across all axes	Acceleration, (x,y,z)∈{V-AP-ML}	$\max\left(\mathrm{RMS}\;\left(x_{\mathrm{RL}}{,y}_{\mathrm{RL}}{,z}_{\mathrm{RL}}\right),\mathrm{RMS}\;\left(x_{\mathrm{LL}}{,y}_{\mathrm{LL}}{,z}_{\mathrm{LL}}\right)\right)$ Gyroscope, (x,y,z)∈{V-AP-ML}
1	$\frac{\max\left(\mathrm{RMS}\;\left(x_{\mathrm{RL}}{,y}_{\mathrm{RL}}{,z}_{\mathrm{RL}}\right),\mathrm{RMS}\;\left(x_{\mathrm{LL}}{,y}_{\mathrm{LL}}{,z}_{\mathrm{LL}}\right)\right)\;}{\mathrm{RMS}\;\left(x_{\mathrm{back}}{,y}_{\mathrm{back}}{,z}_{\mathrm{back}}\right)}$	Legs: Gyroscope, (x,y,z)∈{V-AP-ML} Back: Acceleration, (x,y,z)∈{V-AP-ML}	
1	$\frac{\max\left(\mathrm{RMS}\;\left(x_{\mathrm{RL}}{,y}_{\mathrm{RL}}{,z}_{\mathrm{RL}}\right),\mathrm{RMS}\;\left(x_{\mathrm{LL}}{,y}_{\mathrm{LL}}{,z}_{\mathrm{LL}}\right)\right)\;}{\mathrm{RMS}\;\left(x_{\mathrm{back}}{,y}_{\mathrm{back}}{,z}_{\mathrm{back}}\right)}$	Legs: Gyroscope, (x,y,z)∈{V-AP-ML} Back: Gyroscope, (x,y,z)∈ {V-AP-ML}	
**Frequency domain**			
16	SD(x); SD of the frequency amplitude in specific bands	Acceleration, $x\in \;\left\{\begin{array}{c} V_{0.5–3\;\mathrm{Hz},\;3–8\;\mathrm{Hz}},\\ {\mathrm{AP}}_{0.5–3\;\mathrm{Hz},\;3–8\;\mathrm{Hz}\;}\; \end{array}\right\}$	$\min(\mathrm{SD}\left(x_{\mathrm{RL}}\right),\mathrm{SD}\left(x_{\mathrm{LL}}\right))$ $\max(\mathrm{SD}\left(x_{\mathrm{RL}}\right),\mathrm{SD}\left(x_{\mathrm{LL}}\right))$ Acceleration, $x\in V_{0.5–3\;\mathrm{Hz},\;3–8\;\mathrm{Hz}}$ Gyroscope, $x\in {\mathrm{ML}}_{0.5–3\;\mathrm{Hz},\;3–8\;\mathrm{Hz}}$
24	Peak amplitude of x and its frequency across all frequencies and in specific bands	Acceleration, $x\in \left\{\begin{array}{c} V_{\mathrm{all},\;0.5–3\;\mathrm{Hz},\;3–8\;\mathrm{Hz}},\\ {\mathrm{AP}}_{\mathrm{all},\;0.5–3\;\mathrm{Hz},\;3–8\;\mathrm{Hz}\;}\; \end{array}\right\}$	${\max(x}_{\mathrm{RL}}{,x}_{\mathrm{LL}})$ Acceleration,$\;x\in \;\left\{\begin{array}{c} V_{\mathrm{all},\;0.5–3\;\mathrm{Hz},\;3–8\;\mathrm{Hz}},\\ {\mathrm{AP}}_{\mathrm{all},\;0.5–3\;\mathrm{Hz},\;3–8\;\mathrm{Hz}\;}\; \end{array}\right\}$ Gyroscope, $x\in \;\left\{\begin{array}{c} V_{\mathrm{all},\;0.5–3\;\mathrm{Hz},\;3–8\;\mathrm{Hz}},\\ {\mathrm{ML}}_{\mathrm{all},\;0.5–3\;\mathrm{Hz},\;3–8\;\mathrm{Hz}\;}\; \end{array}\right\}$
3	Entropy of x in specific bands	Acceleration, $x\in {\mathrm{AP}}_{0.5–3\;\mathrm{Hz},\;3–8\;\mathrm{Hz}}$ Gyroscope, $x\in V_{0.5–3\;\mathrm{Hz},\;3–8\;\mathrm{Hz}}$	.
1	Freezing index (x); $\frac{\mathrm{integrated}\;\mathrm{power}\;\mathrm{spectral}\;\mathrm{density}\;\mathrm{in}\;3–8\;\mathrm{Hz}}{\mathrm{integrated}\;\mathrm{power}\;\mathrm{spectral}\;\mathrm{density}\;\mathrm{in}\;0.5–3\;\mathrm{Hz}}$	.	Acceleration, $x\in {\mathrm{AP}}_{\;0.5–3\;\mathrm{Hz},\;3–8\;\mathrm{Hz}\;}$ $\mathrm{freezing}\;\mathrm{index}=\max\left(\begin{array}{c} {\mathrm{freezing}\;\mathrm{index}}_{\mathrm{RL}},\\ {\mathrm{freezing}\;\mathrm{index}}_{\mathrm{LL}} \end{array}\right)$ $\mathrm{freezing}\;\mathrm{index}=\{\begin{array}{cc} 1 & \mathrm{if}\;\mathrm{freezing}\;\mathrm{index}>2.5\\ 0 & \mathrm{else} \end{array}$
1	Total power: the average power of x	While turning: Gyroscope, x∈V Otherwise: Acceleration, x∈AP	.
5	Skewness of x in specific bands	Acceleration, $x\in \left\{\begin{array}{c} V_{0.5–3\;\mathrm{Hz},\;3–8\;\mathrm{Hz}},\\ {\mathrm{AP}}_{0.5–3\;\mathrm{Hz},\;3–8\;\mathrm{Hz}} \end{array}\right\}$ Gyroscope, $x\in {\mathrm{ML}}_{0.5–3\;\mathrm{Hz}}$	.
4	Kurtosis of x in specific bands	Acceleration, $x\in \left\{\begin{array}{c} V_{0.5–3\;\mathrm{Hz},\;3–8\;\mathrm{Hz}},\\ {\mathrm{AP}}_{0.5–3\;\mathrm{Hz},\;3–8\;\mathrm{Hz}} \end{array}\right\}$	.

RL, right leg; LL, left leg; V, vertical *axis*; AP, anterior-posterior *axis*; ML, medio-lateral *axis*; SD, standard deviation; corr, correlation; RMS, root-mean-square.
